# Habitat Protection Approaches Facilitate Conservation of Overlooked Fungal Diversity – A Case Study From the Norwegian Coastal Heathland System

**DOI:** 10.3389/ffunb.2022.886685

**Published:** 2022-05-27

**Authors:** Rakel Blaalid, Marie L. Davey

**Affiliations:** ^1^ Department of Natural History, University Museum of Bergen, Bergen, Norway; ^2^ Norwegian Institute for Nature Research, NINA Bergen, Bergen, Norway; ^3^ Norwegian Institute for Nature Research, Terrestrial Biodiversity Department, Trondheim, Norway

**Keywords:** coastal heathland, mycoflora, conservation, diversity, ericaceous fungi, root associated fungi

## Abstract

European coastal heathlands are distinct ecosystems shaped by land use tradition and they have experienced an 80% area reduction from their historical maximum. These mosaics of mires and wind exposed patches have ericaceous shrub dominated vegetation, and soils within coastal heathlands are characterized by low pH and high levels of recalcitrant debris. Using a culture-based approach with molecular identification of isolates, we characterized root-associated fungal communities of six ericaceous species in eight heathland localities along Norway’s western coast. Site-level alpha diversity ranged from 21-38 OTUs, while the total estimated gamma diversity for culturable heathland root fungi was 190-231 OTUs. Most species recovered are previously reported at low abundance in Norway, suggesting the biodiversity in this community is underreported, rather than novel for science. The fungi recovered were primarily Ascomycota, specifically endophytic *Phialocephala*, and *Pezicula*, and no host specificity was observed in the communities. The fungal communities exhibited high turnover and low nestedness, both between ericaceous hosts and across heathland sites. We observed no spatial patterns in fungal betadiversity, and this heterogeneity may be a product of the unique historic land use practices at each locality creating a distinct mycofloral “fingerprint”. Robust diversity estimates will be key for managing fungal biodiversity in coastal heathlands. Our results indicate that sampling schemes that maximize the number of host plants sampled per site, rather than the number of cultures per plant yield improved alpha diversity estimates. Similarly, gamma diversity estimates are improved by maximizing the total number of localities sampled, rather than increasing the number of plants sampled per locality. We argue that while the current protected status of coastal heathland habitats and restoration efforts have knock-on effects for the conservation of fungal biodiversity, fungi have a vital functional role in the ecosystem and holistic conservation plans that consider fungal biodiversity would be beneficial.

## Introduction

The United Nation 2030 Agenda for Sustainable Development specifically calls for efforts to halt biodiversity loss and species extinction, and to safeguard and conserve key biodiversity areas (United Nations, 2015). Semi-natural landscapes, including coastal heathlands, are among the most endangered habitats in Europe (European Commission et al., 2017; Herzon et al., 2021), and their persistence is dependent on anthropogenic activity. Coastal heathlands stretch along the Atlantic coastline in Western Europe from Northern Portugal to Northern Norway and are maintained by cyclical burning, cutting, and grazing. The people living in these historic areas are unified in terms of land-use practices over space and time (Kaaland, 2012; Siepel et al., 2013) and the coastal-heathland land-use tradition dates back to ca. 5000 years before present (Kaaland, 2012; Hjelle et al., 2018), underpinning their importance as distinct ecosystems, but also their societal value (Siepel et al., 2013; Herzon et al., 2021). As a habitat type, coastal heathlands are characterized by being treeless, shrub-dominated areas, typically with nutrient poor soils. Biodiversity research and conservation in coastal heathlands has been strongly biased towards plant diversity (Vandvik et al., 2005; Måren and Vandvik, 2009; Calvo et al., 2012; Fagúndez, 2013; Velle et al., 2014; Velle and Vandvik, 2014). Some insect groups including beetles (Schirmel, 2010; Schirmel and Buchholz, 2011; Bargmann et al., 2015) have received attention, however, taxonomic groups including most invertebrates, fungi, and other microorganisms have largely been overlooked or poorly circumscribed despite their functional importance to the ecosystem. Today, the coastal heathlands are threatened (Fagúndez, 2013), and their area has been reduced by 80% due to regrowth as a consequence of land use change, urbanization and long-range transported nitrogen deposition (Bobbink et al., 2010; Bahring et al., 2017). About one third of the total area of European coastal heathland habitat occurs in Norway, meaning national level protection and conservation strategies by the Norwegian authorities significantly impact the global status of this habitat type. This has led to government mandates protecting coastal heathlands in Norway: coastal heathlands have been declared a prioritized nature type specifically protected by law (Ministry of Climate and Environment, 2009) and have been placed on the national habitat red list (Norwegian Biodiversity Information Centre, 2018) making them a focus for conservation and restoration efforts, in line with the UN sustainable development goals.

There has been increasing focus on the restoration of heathlands within Europe (Diaz et al., 2006; de la Pena et al., 2012; Blindow et al., 2017; Omand et al., 2018; van der Bij et al., 2018; Radujković et al., 2020). Plant-soil feedback loops have been established as drivers of plant diversity, abundance, and succession (Bardgett and van der Putten, 2014) and heathland restoration experiments have underpinned the importance of plant-fungal interactions to achieve restoration success (de la Pena et al., 2012; van der Bij et al., 2018; Radujković et al., 2020). The currently understudied fungal diversity in European coastal heathlands is therefore expected to play an important role in terms of conservation and restoration of these landscapes. However, fungi as a group have been ignored in action plans for maintaining and restoring this particular nature type (Olmeda et al., 2020) and the group receives limited conservation effort worldwide (Griffith, 2012; Heilmann-Clausen et al., 2015; Gonçalves et al., 2021) despite playing important roles in ecosystem function and moderating essential ecosystem services. This can be attributed to a combination of decreasing competence in fungal species identification within the biological community (Grube et al., 2017) and the inconspicuousness, high diversity, and ephemeral occurrence of many of these organisms. Together, these factors make it difficult to monitor and manage fungi, so they are frequently excluded from action plans for maintaining and restoring specific nature types. However, in Fennoscandia a long fungal taxonomic tradition has led to comparatively well-developed fungal conservation efforts (Dahlberg et al., 2010), with some countries having national red lists of fungi or even action plans to protect specific fungal habitats (Dahlberg et al., 2010; Heilmann-Clausen et al., 2015). In light of this, coastal heathlands in Norway provide a good framework for exploring holistic management strategies focused on total biodiversity that include fungi, as the habitat is already of high priority for conservation and the mechanisms for conservation of fungal biodiversity are comparatively well developed.

Coastal heathland vegetation is dominated by members of the family Ericaceae, which are known to host a diverse array of parasitic, pathogenic, and symbiotic fungi (Walker et al., 2011) in addition to providing litter inputs to the saprophytic fungal community (Berendse, 1998). Endophytic fungi are known to include both latent saprotrophs and pathogens but can also provide a variety of benefits to their hosts including increased stress tolerance and anti-herbivory properties. Among those symbiotic fungi known from ericaceous hosts are ericoid mycorrhiza (ERM) which form a nutrient exchange symbiosis. Here, the fungal partner receives photosynthates from the host while providing nutrients, particularly nitrogen, in return. ERMs are more efficient at acquiring nitrogen than their host plants (Smith and Read, 2008), exploiting various nitrogen sources including amino acids and peptides, making them crucial for host persistence and survival within the nutrient poor edaphic environments of coastal heathlands. These fungi have a complex genomic composition and display versatile life strategies (Perotto et al., 2018). They can increase host tolerance for heavy metals (Straker, 1996; Smith and Read, 2008) and can affect flower phenology and synchrony which in turn affects plant-pollinator interactions (de la Pena et al., 2012), thereby directly influencing host survival and reproductive success. Finally, ERM display trophic lability and can produce extracellular enzymes that degrade complex organic molecules, making them nutrient mediators with an important role in soil carbon cycling (Talbot et al., 2008). Fungi forming ERM and ericaceous root endophytes are demonstrated to be non-host specific (Straker, 1996; Kjoller et al., 2010; Walker et al., 2011; Vohník, 2020) and taxonomically limited (Monreal et al., 2000; Dighton, 2009). Nevertheless, the heathland mycoflora is clearly distinct from surrounding areas that are typically covered by forests (Bougoure et al., 2007; Collier and Bidartondo, 2009). Coastal heathland soils differ significantly in pH, moisture, and organic matter content from these neighboring habitats (Wilson and Puri, 2001; Chapman et al., 2003), and these edaphic factors are all known to influence the mycoflora. In particular, pH is a well-known driver of fungal beta diversity (Tedersoo et al., 2020) which over time shapes the fungal community (Geml et al., 2014; Radujković et al., 2020). Not surprisingly, turnover is observed within the fungal community along forest to heathland gradients (Anderson et al., 2003; Read et al., 2004; Bougoure et al., 2007), with increasing dominance of Ascomycota in the heathlands due to the absence of trees (Anderson et al., 2003; Collier and Bidartondo, 2009).

In this study, we characterized the root associated fungal community in eight Norwegian coastal heathland systems. More specifically we examined the richness, and community composition and structure between (I) host plants and (II) localities. Lastly, we discuss the knock-on effect of habitat protection for fungal conservation, and how fungal diversity estimates, and sampling strategies can be implemented in conservation planning within vulnerable habitats.

## Materials and Methods

**Figure 1:**
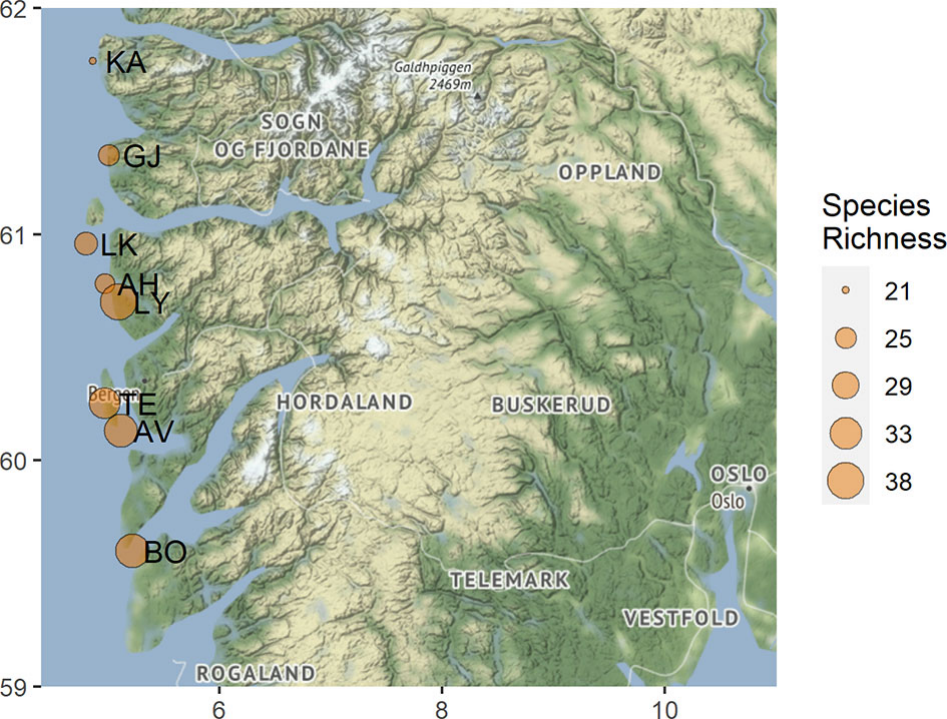
Map displaying the visited coastal heathland localities within western Norway. The observed OTU richness for each locality is indicated by the point size and the abbreviated locality name is found to the right of each point. AV, Austevoll; AH, Austrheim; BO, Bømlo; GJ, Gjerdevik; KA, Kalvåg; LK, Litle Kvernøy; LY, Lygra; TE, Televåg.

During the summers of 2018-19, root-associated fungal communities on ericaceous hosts were investigated in eight Norwegian coastal heathland localities (**Figure 1**). The sampled heathlands spanned 2.17 degrees of latitude and experience similar climatic conditions, with mean annual temperatures ranging from 6.6 to 7.7°C and mean annual precipitation ranging from 1614 to 2202 mm (**Table 1**). Although there was clear variation in the management regimes and plant communities across the eight localities, they were all intact heathlands. Some of the localities like Lygra were clearly still managed using the old farming practices, including burning, cutting and grazing, while other, such as Bømlo, showed signs of secondary succession from *Juniperus commune* and *Betula pubescens* (Blaalid and Davey. Pers obs 2018). All localities were still grazed, and thus managed to some extent. Older surveys have recorded the floral communities at all localities included in this study, however, data on management such as burn cycles and grazing intensities are missing (Fremstad et al., 1991). Our study did not include plant diversity as a parameter for fungal diversity, and we did not record plant diversity at localities, nor within the plots where we sampled host plants. A combined culturing and DNA-barcoding approach was used to characterize the fungal communities associated with the roots of the most dominant plant taxa in the landscape: the ericaceous species; *Calluna vulgaris*, *Erica tetralix*, *Vaccinium myrtillus*, *V. uliginosum* and *V. vitis-idea*. Within four localities, we collected additional specimens of the host *Erica cinerea*, as this species has a narrower niche, and may therefore harbor a distinct fungal community. At each site, plants were collected centrally: at least 25 m from adjoining forest borders or shorelines with at least 10 m between individual plants. A single adult, healthy representative plant of each host species was dug up with approximately a 2 L volume of soil surrounding an intact portion of the root system and placed in a sealed plastic bag that was kept cool (<8°C) during transport to the laboratory facilities.

**Table 1:** Overview of the locality position, climate, culture richness, species richness and richness estimates across the eight Norwegian coastal heathland localities.

Locality	Lon.	Lat.	MAT	MAP	No. Plants	No. Cultures	SObs	SEstC	SEstP
Austevoll	5.11	60.13	7.7	1864	5	86	34	69.7 ± 23.6	79.1 ± 26.4
Austrheim	4.97	60.78	7.7	2071	5	47	24	27.6 ± 3.2	52.9 ± 19.3
Bømlo	5.22	59.60	7.5	1614	6	132	35	72.2 ± 29.5	95.0 ± 39.5
Gjerdevik	5.01	61.35	7.1	2202	5	111	25	45.1 ± 20	61.1 ± 25.2
Kalvåg	4.86	61.77	6.6	2202	6	101	21	26.9 ± 6.4	28.6 ± 5.8
Litle Kvernøy	4.81	60.96	7.6	2068	5	123	26	33.9 ± 7.4	106.0 ± 67.5
Lygra	5.10	60.70	7.7	2095	5	103	38	53.8 ± 10.5	72.8 ± 18.5
Telavåg	4.97	60.25	7.6	1894	6	84	32	45.8 ± 9.8	102.4 ± 45.5

Lon: longitude, Lat: latitude, MAT: mean annual temperature (°C), MAP: mean annual precipitation (mm), SObs: observed species richness, SEstC: asymptotic estimate of species richness with additional cultures per host, SEstP: asymptotic estimate of species richness with additional hosts collected at each locality.

### Culturing and Morphotyping

Root associated fungi were isolated from the host plants following the protocol described by Hambleton and Currah (1997) with some modifications. Root systems were first thoroughly washed in tap water to remove soil, debris, foreign roots, and ericaceous roots not physically connected to the host plant. Healthy, fine hair roots (as described by Vohník, 2020) were excised from the root system using scissors. The hair roots were divided into two subsamples, which were subjected to a weak or a stringent surface sterilization protocol. Two surface sterilization protocols were used, as Smith and Read (2008) indicate that even short duration chemical surface sterilization can kill some fungi growing within plant cells. Half of the fine roots were subjected to one minute surface sterilization in 1% NaOCl, while the remaining were subjected to a 3-minute surface sterilization regime, as recommended by Hambleton and Currah (1997). For each surface sterilization treatment, roots were then cut into 1-3 mm pieces using sterile forceps and a scalpel, and 8-10 fragments were placed on two different isolation media 100 ug/mL tetracycline: Oatmeal Agar (OA) and Modified Melin-Norkrans (MMN). The resulting four isolation plates from each plant were stored in a dark climate chamber for 30 days at 14°C. Hyphae emerging from the root fragments were then aseptically transferred to OA or MMN to generate pure cultures of individual fungal species. Subcultures were incubated for 60 days at 14°C in a dark climate chamber. Those subcultures that successfully grew were first grouped by locality and then further grouped into morphotypes within each locality based on colony morphology (margin, texture, colony color, secreted pigments etc.). We avoided creating morphotypes spanning localities and refrained from grouping cultures with any ambiguities. We sampled material for both DNA extraction and permanent storage at -80 degrees in 20% glycerol (from fungal material from each of the cultures using a clean sterile pipette tip. The voucher material for permanent storage is kept within the facilities at the University Museum of Bergen.

### DNA Extraction, PCR and Sequencing

The fungal material was crushed in CTAB buffer for 2 minutes at 30 Hz using a Qiagen Retch tissue lyser. The DNA was then extracted using a CTAB approach (Murray and Thompson, 1980) with modifications (Gardes and Bruns, 1993). DNA isolates were cleaned using a column cleanup kit (VWR pregold Cyclepure) and sent to Barcode of Life initiative (BOLD) (Ratnasingham and Herbert, 2007) for sequencing the internal transcribed spacer region (ITS), which is the chosen barcoding marker for fungi (Schoch et al., 2012). The sequences were generated by using the primer pair ITS1-F and ITS4 (White et al., 1990; Gardes and Bruns, 1993) and were sequenced bidirectionally. All sequences are stored within the BOLD system database (Ratnasingham and Hebert, 2013) including a reference picture, which are freely available. The sequences can be accessed by searching by either the project code “FINCH”, or individually by sequence ID (**Table S1**). We then annotated our sequences using the UNITE database as a reference (Nilsson et al., 2018; Kõljalg et al., 2020), and all sequences identified to a unique species hypothesis in the UNITE database (using a 95% identity threshold as described by U’ren et al., 2009) were treated as belonging to a single OTU (**Table S1**). Sequences were assigned membership to functional guilds using the FunGuildR package for R based on the FUNguild database (Nguyen et al., 2016) and each sequence’s best BLAST match to the UNITE database with percent identity >95%.

### Statistical Analyses

All statistical tests and figures were generated in the R statistical environment (R Core Team, 2018). The *iNEXT* package (Hsieh et al., 2016) was used to generate alpha diversity estimates for i) the site level based on number of cultures and number of plants sampled and ii) the host level based on both number of cultures and number of sites sampled (**Table 1**). In addition, gamma diversity was estimated for the coastal heathland landscape as a whole, based on both number of cultures and number of sites. Sorensen’s beta diversity was calculated pairwise between each of the sampled root systems using the betapart package (Baselga and Orme, 2012), and subsequently partitioned into its nestedness and turnover components. For each of these three measures, we examined i) the distance decay relationship with the geographic distance between sites and ii) the differences between the categories: between hosts-within sites, between hosts-between sites, and within host - between sites. Distance decay relationships were tested using linear models, as the data were normally distributed according to Shapiro-Wilkes tests, while between host and between site differences were not-normally distributed and assessed using a Kruskal-Wallis test with a *post-hoc* Dunn test to identify differences between groups. Patterns in beta diversity were also explored in an NMDS ordination of those root systems for which >7 cultures were generated. The ordination structure was calculated using the *metaMDS* function in the *vegan* package (Oksanen et al., 2020) on a Bray-Curtis dissimilarity matrix with two dimensions and a maximum of 500 random starts.

## Results

We attempted isolation of 1196 fungal colonies from the 160 isolation plates that were established. Of these, 145 were discarded after failure to grow, obvious signs of aerial contamination, or indications that the culture was mixed with multiple species. The remaining 1051 isolates were grouped into morphotypes, yielding 456 morphological entities. A representative culture was sequenced from each morphotype, and a total of 386 high quality sequences were generated from these cultures, giving a morphotype sequencing success rate of 85%. In total, 787 cultures were identified representing 125 unique species hypotheses in the UNITE database, hereafter simply referred to as OTUs.

**Figure 2:**
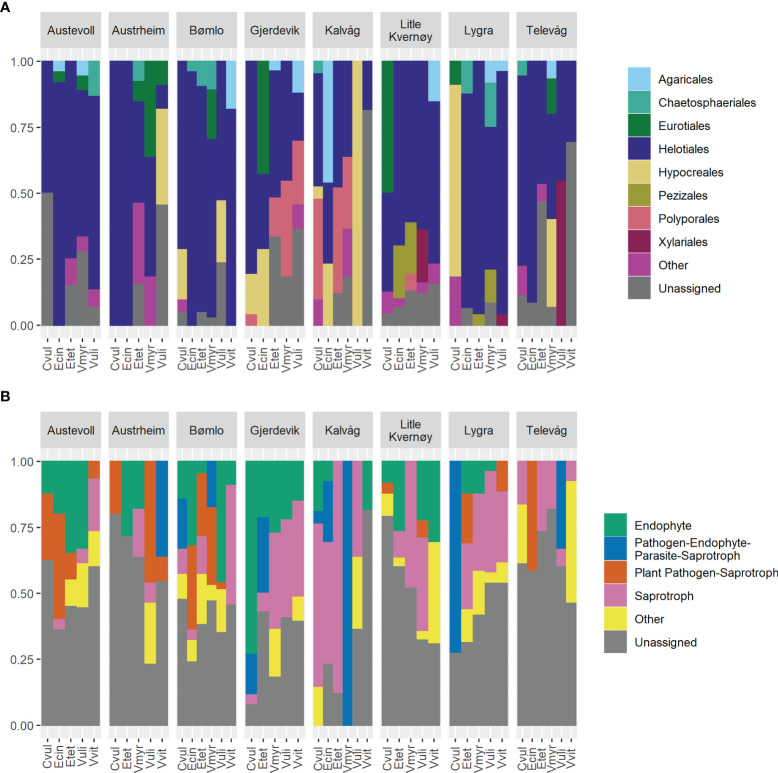
The observed gamma diversity (total OTU richness) across the heathland sites inventoried, with estimates of total richness based on further sampling of **(A)** additional sites and **(B)** additional host plant root systems.

There was no significant difference (p=0.2448) in the number of OTUs recovered per site with weak versus stringent surface sterilization regime and compositional differences between the two methods were of a smaller magnitude than those observed between sites (data not shown). Over 75% of the root-associated cultures were members of the Ascomycota, primarily among the Leotiomycetes and Sordariomycetes. At the order level, 87% of the cultures were successfully assigned taxonomy and Helotiales was the dominant group in all hosts and localities (**Figure 2A**) *Phialocephala* was the most commonly isolated genus, representing 15% of the cultures generated, and was found in association with all hosts and at every locality with the exception of Telavåg. Members of the Hyaloscyphaceae (7% of cultures), and the genera *Penicillium* (7%) and *Pezicula* (6%) were also frequently recovered. The cultures recovered largely represented endophytes, plant pathogens, and saprophytes (**Figure 2B**), although it must be noted that 43% of cultures could not be assigned to a guild using the FunGuild database.

**Figure 3:**
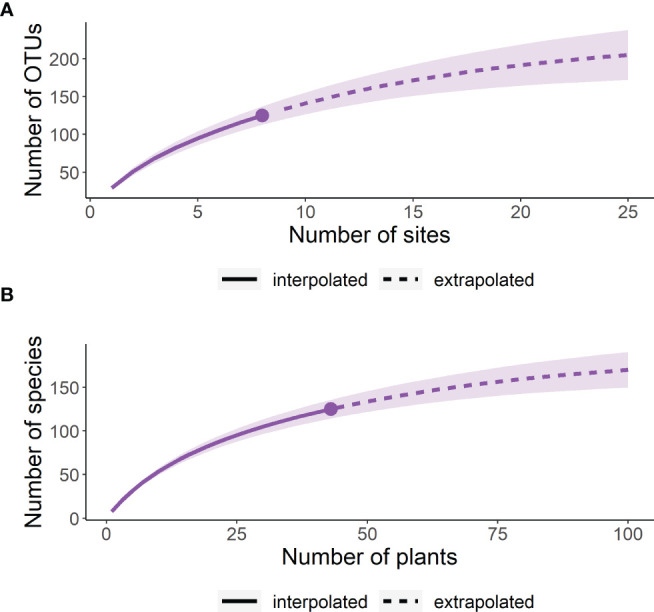
Taxonomic diversity **(A)** and distribution of functional guilds **(B)** across hosts and sites. The proportion of cultures identified to each order **(A)** or guild **(B)** is shown for each host at each site.

**Figure 4:**
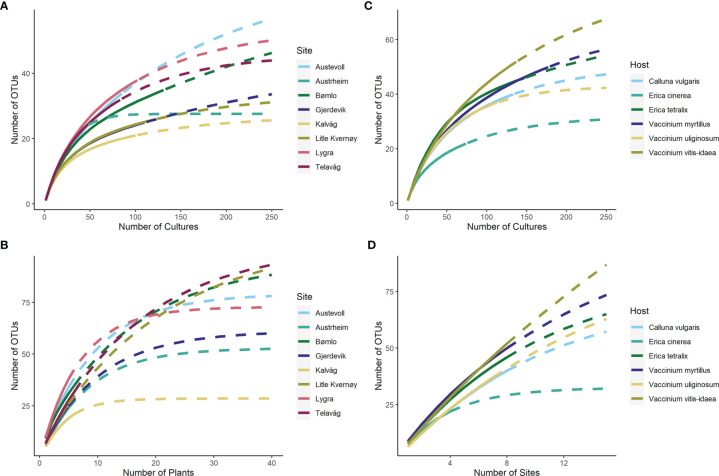
OTU accumulation curves showing the observed and estimated alpha diversity associated with each site **(A, B)** and each host species **(C, D)**. Asymptotic estimates of total diversity are calculated based on sampling of additional cultures **(A, C)**, additional host plants **(B)** or additional sites **(D)**.

The gamma diversity of root associated fungi across the coastal heathland landscapes surveyed was at least 125 OTUs (**Figure 3**). Estimates of the total possible gamma diversity that could be recovered by surveying either more plants (**Figure 3B**) or more sites (**Figure 3A**) ranged from 190-231 OTUs. The alpha diversity observed across hosts at the individual sites ranged from 21 to 38 OTUs (**Figure 4A**), with estimates of total richness of up to 106 species per site given the sampling of additional plants (**Figure 4B** and **Table 1**). The alpha diversity associated with each host species was very similar, ranging from 37 to 52 OTUs for those hosts collected at all sites (**Figure 4C**). However, estimated asymptotic OTU richness with further collection of the hosts at additional sites was more variable, ranging from 73 OTUs for *Calluna vulgaris* to 229 OTUs for *Vaccinium vitis-idaea* (**Figure 4D** and **Table 2**).

**Table 2:** Observed and estimated species richness associated with six hosts collected across eight Norwegian coastal heathlands.

Host	No. Cultures	No. Localities	Sobs	SEstC	SEstP
*Erica tetralix*	148	8	46	68.3 ± 16.0	83.3 ± 18.1
*Erica cinerea*	75	4	22	32.0 ± 8.9	32.5 ± 7.6
*Vaccinium myrtillus*	178	8	50	68.1 ± 10.6	106.7 ± 27.1
*Vaccinium vitis-idaea*	138	8	52	85.6 ± 17.9	229.1 ± 96.2
*Calluna vulgaris*	134	8	40	50.8 ± 7.6	73.5 ± 17.0
*Vaccinium uliginosum*	114	7	37	43.0 ± 4.8	101.3 ± 36.1

SObs: observed species richness, SEstC: asymptotic estimate of species richness with additional cultures per plant, SEstP: asymptotic estimate of species richness with additional sampling of host plants.

**Figure 5:**
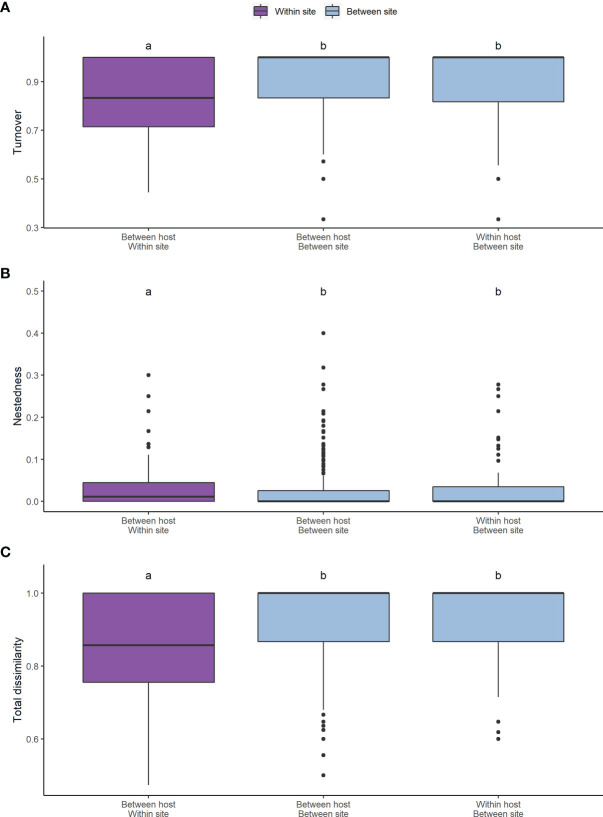
Pairwise Sorensen’s index for betadiversity across sites and ericaceous hosts. In addition to **(C)** the total pairwise dissimilarity (p<<0.001), the **(A)** turnover (p<<0.001) and **(B)** nestedness (p=0.011) components of betadiversity are shown. Significantly different groupings identified by a Dunn test are indicated by letters over the categories.

**Figure 6:**
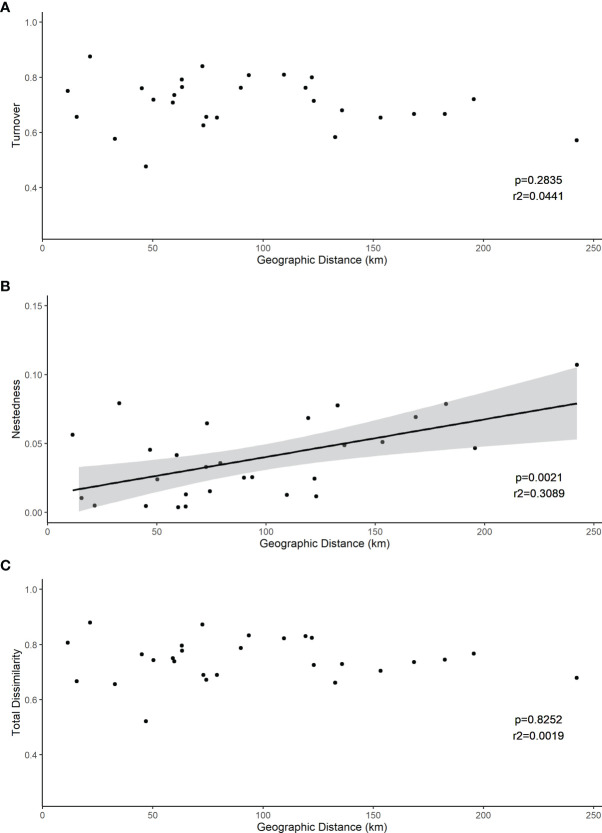
Between-site distance decay relationships between betadiversity and geographic distance between sites. Both **(C)** total dissimilarity (Sorensen index, p<<0.001) and its component **(A)** turnover (p<<0.001) and **(B)** nestedness (p=0.0) are shown.

The root associated fungal communities were highly heterogeneous, exhibiting high turnover and low nestedness, both between ericaceous hosts and across heathland sites (**Figure 5**). Nevertheless, total dissimilarity and turnover between the root fungal communities were significantly lower (both p<<0.001) at “between hosts- within site” compared to “within host - between sites” or “between hosts - between sites” (**Figure 5**). Concomitantly, nestedness was significantly higher (p=0.011). However, we observed no spatial autocorrelation at larger scales and neither beta diversity between the heathland sites, nor its component nestedness and turnover, exhibited a classic distance decay relationship with increasing distance between sites (**Figure 6**). Ordination analysis of those root systems for which >8 cultures were obtained and successfully sequenced showed weak differences among localities (p=0.014) and no significant structuring by host species (p=0.256) (**Figure S1**).

## Discussion

### Taxonomy and Diversity

The observed dominance of Ascomycota within the coastal heathland system is consistent with previous investigations of the heathland mycoflora (Anderson et al., 2003; Bougoure et al., 2007). We frequently encountered *Phialocephala* species within the root systems and this genus is a commonly isolated dark septate endophyte of ericaceous hosts (Grünig et al., 2008; Jumpponen and Trappe, 2008), as are the *Cryptosporiopsis* anamorphs of *Pezicula* (Sigler et al., 2005; Yang et al., 2018). *Phialocephala fortinii* was frequently recovered across hosts and sites and this species is known to increase host above- and below-ground biomass, as well as host N- and P-content (Newsham, 2011). Given the prevalence of this species complex and its impacts on host growth and reproduction, *P. fortinii* is likely a vital species in terms of host persistence and function in coastal heathlands. Several well-known ERM species (Perotto et al., 1996; Monreal et al., 2000; Smith and Read, 2008) were also isolated, including members of the Hyaloscyphaceae, *Meliniomyces, Oidiodendron* and *Rhyzoscyphus*. *Mycena* was the most common basidiomycete, and this genus has recently been demonstrated to have functional lability with the capacity to colonize living plant roots (Thoen et al., 2020). Notably, we did not recover Sebacinales within the host mycoflora. These taxa are frequent, culturable associates of ericaceous plant roots (Selosse et al., 2007; Vohnik et al., 2016; Weiß et al., 2016) and have been reported as “ERM like” (Selosse et al., 2007; Vohnik et al., 2016). The taxonomic entities within our study are mostly known from Norway in only sporadic reports both in GBIF and NBIC, however, they are frequently reported in culture- and molecular-based surveys of soil and root fungal biodiversity (Vrålstad et al., 2002a; Vrålstad et al., 2002b; Blaalid et al., 2012; Blaalid et al., 2013). This would suggest that the biodiversity in the ericaceous root-associated fungal community is underreported, rather than novel for science.

Estimates of the total ericaceous root-associated fungal diversity in Norwegian coastal heathlands are as high as 231 OTUs. However, it must be noted that due to methodological bias, this estimate is unlikely to reflect the true total fungal diversity associated with ericaceous roots in this habitat. Numerous studies have documented a taxonomically diverse array of fungi that are not readily cultured and rarely or never detected in culture-based studies (Arnold et al., 2007; Higgins et al., 2011; Høyer and Hodkinson, 2021). Metabarcoding based culture-independent studies of ericaceous hosts (*Vaccinium calycinum:* Leopold et al., 2021, *Cassiope tetragona*: Lorberau et al., 2017) report more than 600 OTUs per host, indicating that the true root-associated fungal diversity in Norwegian coastal heathlands is likely much higher than what is reported here.

### Community Heterogeneity

The ericoid root-associated communities in coastal heathlands exhibited both low nestedness and high rates of turnover between sites. This high degree of between-site heterogeneity is consistent with findings from other inventories of heathland fungal communities (de la Pena et al., 2012; Macia-Vicente and Popa, 2022). As observed by Macia-Vicente and Popa (2022), there was no discernible distance decay relationship in community composition, and the Norwegian heathland communities each had a distinct mycofloral “fingerprint” that likely reflects local stochastic processes that create a high degree of endemism within ericaceous root fungi. Coastal heathland habitats are formed across centuries of anthropogenic activity (Hjelle et al., 2010; Kaaland, 2012), including prescribed burning, turf cutting, and grazing, all of which shape the habitat’s vegetation (Vandvik et al., 2005; Måren and Vandvik, 2009; Velle and Vandvik, 2014; Bargmann et al., 2015). The unique land use history of each coastal heathland likely shapes the characteristic fungal community found in each location as well (Hazard et al., 2014). For example, grazing intensity and grazer identity can impact both soil fungal community diversity and biomass (van der Heyde et al., 2017; Eldridge and Delgado-Baquerizo, 2018; Xun et al., 2018; Dudinszky et al., 2019). However, the direct effects on plant associated mycoflora of the low to moderate grazing pressure that most coastal heathlands experience remains largely unknown. However, studies of ectomycorrhizal fungi suggest that factors other than grazing may be more influential in shaping the plant-associated mycoflora (Dudinszky et al., 2019; Tervonen et al., 2019). For example, coastal heathlands are subjected to periodic burning, and fire is known to affect microbial community composition (Hewitt et al., 2016). Within coastal heathlands, fire disturbance has historically occurred on a 20–30-year cycle (Kaaland, 2012) and these fires reduce nitrogen and phosphorus levels in heathland soils (Green et al., 2013), which in turn is expected to affect fungal community dynamics. Still, little is known about how the mycoflora is influenced by such short cyclic fire events. Studies from natural pyrophilic ecosystems have emphasized the predictable effect of fire on fungal community dynamics (Hopkins et al., 2021), and frequent wildfires are known to promote fire specialists (Hansen et al., 2019). However, coastal heathlands are relatively “young fire landscapes” and it remains to be elucidated the impact of this frequent fire cycle on the mycoflora. The current study (n=8) lacks the needed statistical power to infer the specific roles of land use, including grazing and burning, on shaping fungal communities. Nevertheless, we argue that the highly heterogenous fungal communities we observe in Norway’s coastal heathlands are likely a product of the unique historic land use practices at each locality (Hazard et al., 2014; Tervonen et al., 2019).

Despite identifying a unique mycoflora at each coastal heathland site, we failed to detect signs of host-specificity among the ericoid root-associated fungi surveyed here. The plants investigated hosted similar numbers of fungal species irrespective of host identity, and many of the fungal species were found in association with multiple ericaceous hosts. Previous comparisons of culture-dependent and culture-independent methods for characterizing ericaceous root-associated fungal communities indicate that while the two different methods may recover different species, they detect the same biological patterns (Walker et al., 2011). This suggests the absence of host specificity we observe here is not simply a product of bias towards only those fungi in the root systems that are culturable. Little community divergence between ERM host taxa has been demonstrated in some systems (Kjoller et al., 2010; Walker et al., 2011), but distinct communities partitioned by host identity have been recovered in others (Bougoure et al., 2007; Ishida and Nordin, 2010). We argue that the high degree of versatility in life strategies exhibited by ERM fungi (Perotto et al., 2018) combined with resource partitioning theory (Bruns, 1995) are factors explaining the high diversity and heterogeneity of fungal communities both between hosts and between sites in coastal heathlands.

### Conservation

Fungal conservation is typically under-prioritized (Heilmann-Clausen et al., 2015; Cao et al., 2021; Gonçalves et al., 2021), and few frameworks exist for the management and monitoring of not only individual fungal species, but fungal communities as a whole. Management authorities frequently use alpha and gamma diversity metrics to identify and prioritize areas for conservation, focusing on biodiversity and species richness both at the local and landscape levels. However, there is an increasing body of work that suggests beta diversity metrics can also be useful parameters to assess drivers of community assembly (Anderson et al., 2011; Chiantore et al., 2018) and function as a tool in conservation (Bush et al., 2016; Socolar et al., 2016). In order for fungi to be included in holistic management strategies that focus on total biodiversity, we therefore require well developed protocols for assessing fungal biodiversity that generate robust alpha and beta diversity estimates. Here, standardized monitoring programs will yield important data for capturing such diversity metrics Haase et al., 2018). Despite generating more than 100 cultures for many of the sites examined here, species accumulation curves did not approach saturation for the individual localities nor the total gamma diversity of the coastal heathland landscape. Extrapolated alpha diversity estimates suggest that on a locality level, further recovery of biodiversity would most efficiently be made by increasing the number of plants sampled per locality rather than increasing the number of cultures generated per root system. On a landscape level, gamma diversity estimates would be improved by increasing the number of sites sampled, rather than by increasing the number of plants sampled. Based on these results, we recommend that when assessing biodiversity in coastal heathlands, sampling strategies should be designed to maximize the number of hosts sampled at individual sites in order to provide robust alpha diversity estimates, and to maximize the number of localities sampled for providing the best possible estimate of gamma diversity at the landscape level. This study provides base knowledge for how sampling strategies can be designed to provide the reliable biodiversity estimates that are needed to allow the incorporation of fungal biodiversity into conservation strategies.

Throughout much of Europe, coastal heathlands are already conserved areas owing to their cultural and historical importance. We argue that protection of these areas for their societal value has the knock-on effect of preserving the unique mycofloral biodiversity present in each coastal heathland. We observe high heterogeneity and substantial species turnover between individual coastal heathlands that was unrelated to geographic distance and speculate this may reflect the unique land-use history at each locality. Thus, protecting individual coastal heathland areas indirectly conserves a unique suite of fungi at each site. Such “In-situ” habitat conservation approaches have been suggested for fungi already in the 1990s (Hawksworth, 1991), and coastal heathlands are a prime example of how this concept can function. Notably, focus on heathland restoration has increased in recent years (de la Pena et al., 2012; van der Bij et al., 2018; Radujković et al., 2020). Such restoration will have knock-on effects of conserving such frequently overlooked organismal groups within these habitats, including fungi. Moreover, the success of coastal heathland restoration, as indicated by vegetation establishment, is greatly improved by reinoculation of the belowground community which effectively functions as a conservation measure for fungal biodiversity (van der Bij et al., 2018). However, restoration and conservation of coastal heathlands requires follow-up with a combination of low-intensity measures such as mowing, grazing, burning and regular shrub clearance to maintain the coastal heathland landscape (Blindow et al., 2017; Walmsley et al., 2021), all of which are expected to benefit those species adapted to a semi-natural system, including fungi.

Plant-soil feedback loops have been established as drivers of plant diversity, abundance, and succession (Bardgett and van der Putten, 2014) and heathland restoration experiments have underpinned the importance of plant–fungal interactions to achieve restoration success (de la Pena et al., 2012; van der Bij et al., 2018; Radujković et al., 2020). We argue that in ecosystems like coastal heathlands where fungi play significant ecological roles with effects on multiple trophic levels, holistic conservation and restoration approaches that include action plans for fungi would be beneficial.

### Conclusions

A broad diversity of root-associated fungi was isolated from ericaceous hosts in coastal heathlands, representing primarily Ascomycete endophyte, saprophyte, and ERM fungi. We observed little host specificity among the fungi isolated, as has previously been observed in these communities. The fungal communities at different coastal heathland localities were highly heterogeneous, exhibiting low nestedness and high species turnover, with each locality bearing a unique mycofloral ‘fingerprint’ which we hypothesize may be linked to the unique land-use history at each site, although further research is needed to confirm this. Based on our own attempts to quantify the alpha and gamma diversity in Norwegian coastal heathlands, we recommend that in order to provide robust estimates of alpha and gamma diversity that can be used for management purposes, sampling strategies should aim to maximize the number of plant hosts investigated to improve alpha diversity estimates, and to maximize the number of sites sampled to improve gamma diversity estimates. Finally, existing and future conservation and restoration efforts targeting European coastal heathlands are expected to have knock-on effects, facilitating conservation of fungal biodiversity.

## Data Availability Statement

The datasets presented in this study can be found in online repositories. The names of the repository/repositories and accession number(s) can be found below: https://www.boldsystems.org/index.php/MAS_Management_DataConsole?codes=FINCH, FINCH.

## Author Contributions

RB and MD developed the project, received funding, and did the fieldwork. RB performed the culturing and DNA work. MD performed the statistical analyses. RB and MD wrote the manuscript. All authors contributed to the article and approved the submitted version.

## Funding

This work was funded by the Norwegian Biodiversity Information Centre and as part of the project FINCH-28/17.

## Conflict of Interest

The authors declare that the research was conducted in the absence of any commercial or financial relationships that could be construed as a potential conflict of interest.

## Publisher’s Note

All claims expressed in this article are solely those of the authors and do not necessarily represent those of their affiliated organizations, or those of the publisher, the editors and the reviewers. Any product that may be evaluated in this article, or claim that may be made by its manufacturer, is not guaranteed or endorsed by the publisher.

## Supplementary Material

Supplementary Figure 1 Ordination diagrams showing the relationship between all root systems for which >7 cultures were identified color-coded by **(A)** host (p=0.256) and **(B)** site (p=0.014).
