# Supplementary figures and images for: Habitat Protection Approaches Facilitate Conservation of Overlooked Fungal Diversity – A Case Study From the Norwegian Coastal Heathland System
Source: Front Fungal Biol. 2022 May 27;3:886685. doi: 10.3389/ffunb.2022.886685 (PMC10512255; doi:10.3389/ffunb.2022.886685)

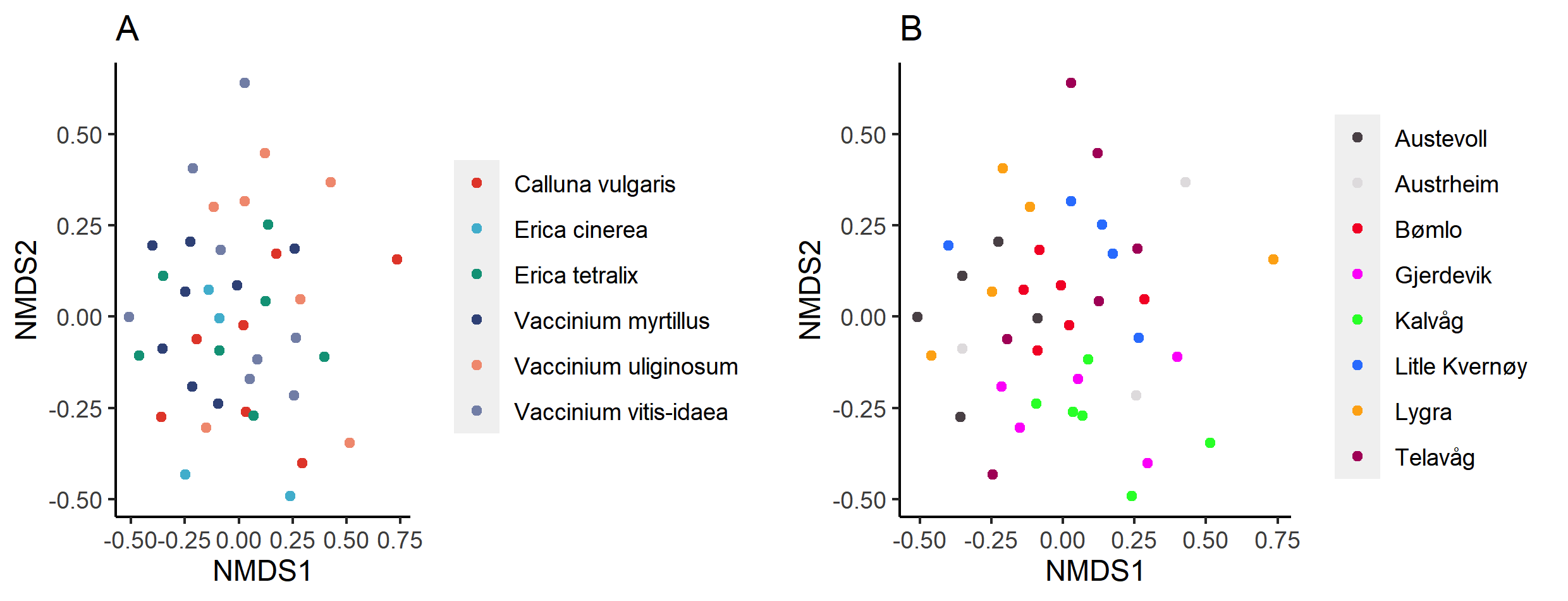

Supplement: Supplementary Figure 1 — Ordination diagrams showing the relationship between all root systems for which >7 cultures were identified color-coded by (A) host (p=0.256) and (B) site (p=0.014). [file Image_1.tiff]
